# Validation of the Malay Translation of Drug Attitude Inventory

**DOI:** 10.7759/cureus.60715

**Published:** 2024-05-20

**Authors:** Huey Jing Renee Tan, Shiao Ling Ling, Norashikin Khairuddin, Arunah Sanggar, Wan Yi Lim, Mahmoud Danaee, Norliza Bt Chemi

**Affiliations:** 1 Department of Psychiatry and Mental Health, Hospital Kajang, Ministry of Health, Kajang, MYS; 2 Department of Psychiatry and Mental Health, Hospital Raja Permaisuri Zainab II, Ministry of Health, Kota Baru, MYS; 3 Department of Social Preventive Medicine, Faculty of Medicine, University Malaya, Kuala Lumpur, MYS

**Keywords:** psychotropic medication, medication adherence behavior, schizophrenia, compliance, drug attitude

## Abstract

Introduction: The Drug Attitude Inventory 9 (DAI-9) is a nine-item self-rated questionnaire. The questionnaire assessed positive and negative attitudes of patients toward taking medication, presence of medication side effects and perceived autonomy in treatment decision.

Aim*: *This study aimed to validate the psychometric properties of the Malay translation of Drug Attitude Inventory 9 (MDAI-9).

Method: DAI-9 was translated from English to Malay via forward and backward translation process to produce MDAI-9. MDAI-9 was then validated on patients with psychosis who were attending psychiatry out-patient clinics.

Results*: *There were 54 participants in this study. The subscale (attitude towards psychotropic medications) has a Cronbach’s α of 0.93, whereas the subscale that assesses the presence of side effect problems has a Cronbach’s α of 0.86*. *Exploratory factor analysis supported a two-factor model. Kaiser-Meyer-Olkin's measure of sampling adequacy was 0.64 and Bartlett’s test of sphericity was significant (​​​​​X^2^^ ^_(36)_ = 281.8, *p *<0.001).

Conclusion:In conclusion, MDAI-9 is reliable and valid.

## Introduction

Antipsychotic medication has been the mainstay of treatment for individuals living with schizophrenia [1]. Treatment guidelines advocated the continuous use of antipsychotics even after remission of symptoms for relapse prevention [2]. However, non-compliance to treatment is a chronic debilitating issue in the long-term management of individuals with schizophrenia [3]. Various studies reported that non-compliance to treatment varies from 4-72% [4]. Treatment compliance was defined as accepting and following medical advice and recommendations [3]. Non-compliance was defined as either not using or irregular use of medications and failing to follow through with clinic appointments [5].

Patient’s attitudes toward medication compliance have been identified as one of the factors associated with drug discontinuation. Identifying early indicators and implementing safe and effective methods to ensure compliance is important. Evaluation of compliance with treatment can be achieved via direct observation, medication pill count, serum level measurements, information from caregivers, clinical observation, and via self-reported questionnaires [6-7]. The use of self-report questionnaires provided a convenient and cost-effective method for the evaluation of medication compliance.

Many questionnaires are available to measure compliance with medication and attitude towards medications. Drug Attitude Inventory (DAI-30) was developed by Hogan et al. in 1983 to assess the attitude towards medication [8]. A shorter version of the scale, the DAI-10, has the predictive accuracy of the full instrument. However, there was a psychometric flaw in the DAI-10: six positively phrased items and four negatively phrased items [9-10]. Three of the negatively phrased items referred to side effects, whereas, the positively phrased items referred to symptom reduction, which creates a confounding problem [9]. The DAI-9 has two subscales, namely the positive and negative subscales and one orphan item. The positive subscale referred to the symptoms alleviating effects of medication whereas the negative subscale referred to the side effects of medication. Orphan item 3 assessed the perceived lack of control to medication [10].

The aim of this study is translating the existing DAI-9 questionnaire into Bahasa Malaysia for the purpose of easing its usage by the locals in the country. The validity and reliability of the scale was then tested in a population of patients with schizophrenia in a local public hospital setting.

## Materials and methods

Translation process

DAI-9 was translated from the source language to Bahasa Malaysia via a forward and backward translation process. Permission to translate DAI-9 was obtained from the author. The original DAI-9 was translated into Malay by a group of psychiatrists who are proficient in both languages to produce MDAI-I. MDAI-I was then translated back to English by another team consisting of a psychiatrist, psychiatry registrar, and psychiatry medical officer to produce MDAI-II. MDAI-II was compared with the original DAI-9. Further editing by the team was done to produce MDAI-III. MDAI-III was pilot-tested on 30 participants who attended a psychiatry clinic in a local hospital. The understanding of the participants on each item was checked to ensure that the semantic meaning of items in the scale was retained. This process produced the final version of MDAI-9.

Data collection process

Participants of this validation study were identified via systematic random sampling. Adult patients diagnosed with brief psychotic disorder, schizophreniform, schizophrenia or schizoaffective disorder based on diagnostic criteria of Diagnostic Statistical Manual 5 were included in the study. Those who were not able to read or understand Bahasa Malaysia and those diagnosed with neurocognitive disorder, intellectual disability, and suicidal tendencies were excluded from the study. Consent was obtained from all participants prior to participation in the study. Participants were required to fill up the sociodemographic form, MDAI-9 and Medication Adherence Rating Scale-M (MARS-M). Ethical approval was obtained from the Ethics Committee of the National Medical Research Registry (NMRR ID-22-02648-OHA).

Research Instrument

DAI-9 is a nine-item self-rated questionnaire. The items on DAI-9 were constructed based on the understanding that compliance was influenced by the patient's positive and negative attitude toward the medication [10]. It has three factors assessing attitudes towards psychotropic medication, the presence of side effects of medications, and perceived autonomy in decision on medication [10-11]. A positive-sum score of < 11 is defined as a negative attitude to medication, while a negative sum score of > 7 suggests a side effect problem [10]. Each question in the questionnaire had a four-point response scale. The scale ranged from does not agree (1), agree to some extent (2), agree to a large extent (3), and agree fully to the statement (4). The participants were required to choose a response that reflected the degree of agreement with each item.

The Medication Adherence Rating Scale-M (MARS-M) is a seven-item self-rated questionnaire in the Malaysian language to assess medication compliance in psychoses. MARS-M is a questionnaire adapted from the Medication Adherence Rating Scale. It is a reliable and validated tool to assess medication compliance. It has two factors assessing medication compliance behavior, attitude towards medication and subjective experience of side effects [12]. It has internal consistency ranging from 0.78 to 0.84 [12]. The higher the sum score of MARS-M, the more compliant the participant is with the medication prescribed. A score of ≥ 6 indicates good compliance, a score of 1-5 indicates partial compliance and a score of ≤ 1 indicates poor compliance.

Data analysis

Statistical analysis was done using SPSS software, version 22.0 (IBM Corp., Armonk, NY) [13]. Demographic characteristics of the participants were analysed using descriptive statistics. Internal consistency was assessed via Cronbach’s α. Exploratory factor analysis was done using FACTOR, a computer program to fit the exploratory factor analysis model [14]. Exploratory factor analysis with bootstrapping was employed using a polychoric correlation matrix for categorical data. Tetrachoric correlation, a special case of the polychoric correlation was applied as both observed variables were dichotomous. The polychoric correlation was advised when the univariate distributions of ordinal items are asymmetric or with excess of kurtosis. Factor Analysis model for binary variables was applied [15]. The factor structure of the items in the questionnaire was examined using Principal Components Analysis (PCA) and promin rotation which enabled analyses based on a polychoric correlation matrix. Parallel analysis was used to determine the number of factors to retain in the scale. Pearson correlation analysis was conducted to examine the correlation between subscales of MDAI-9 and MARS-M.

## Results

Demographic characteristics

There was a total of 54 participants in the study. Table 1 summarizes the sociodemographic data of the participants. The mean age of participants was 39.17 (SD=11.06).

**Table 1:** Demographic characteristics of participants

Characteristics	N	%
Age		
18 – 19	1	1.85
20 – 29	13	24.07
30- 39	14	25.93
40 – 49	12	22.22
50 – 59	14	25.93
Gender		
Male	25	46.30
Female	29	53.70
Marital Status		
Single	27	50.00
Married	20	37.00
Separated	3	5.60
Divorce	3	5.60
Widow / widower	1	1.90
Highest Education		
Primary	3	5.60
Secondary	33	61.10
Diploma	7	13.00
Degree	9	16.70
Others	2	3.70
Household Income (RM)		
<1000	9	16.7
1000-3999	29	53.7
4000-7999	12	22.2
8000-9999	3	5.6
10000-14999	1	1.9
Occupation		
Professional	4	7.4
Teacher	1	1.9
Businessman	1	1.9
Labourer	2	3.7
Homemaker	11	20.4
Retired	2	3.7
Unemployed	18	33.3
Others	15	27.8

Exploratory factor analysis

Exploratory Factor Analysis (EFA) was applied to determine the factor structure among 9 items of the MDAI-9. Kaiser-Meyer-Olkin's measure of sampling adequacy was 0.64, above the suggested value of 0.6, and Bartlett’s test of sphericity was significant (X2 (36) = 281.8, p <0.001). Proportions of the variance (initial communalities) for each variable were accounted for by all components, and small values (<0.3) indicate variables that do not fit well with the factor solution. In the current study, all initial communalities were above the threshold. All loading factors were above 0.5.

The results of EFA on all nine items extracted two factors based on parallel analysis. The eigenvalues and total variance are explained by the two factors shown in Table 2. The results after Promin rotation showed that the first factor which is related to attitude toward psychotropic medication explained 43.7% of the variance and the second factor which is related to side effects of medication was 22.8% of the variance.

**Table 2:** Factor loadings for nine items of the MDAI-9 MDAI-9: Malay translation of Drug Attitude Inventory

Item	Factor 1	Factor 2
Q1	0.851	-
Q3	0.789	-
Q4	0.847	-
Q6	0.755	-
Q8	0.835	-
Q9	0.772	-
Q2	-	0.860
Q5	-	0.776
Q7	-	0.788
Eigenvalues	4.061	1.922
% of Variance	43.7	22.8

Reliability

The study showed that MDAI-9 has good overall internal consistency (α = 0.7). The internal structure reliability for individual factors of the scale is presented in Table 3. Item 3 assessed perceived autonomy in treatment decisions. Internal consistency was unable to be determined for this factor as it only has one item.

**Table 3:** Internal structure reliability

Factor	Cronbach’s Alpha
Attitude to psychotropic medication (Items 1, 3, 4, 6, 8, 9)	0.93
Side effect problem (Items 2, 5, 7)	0.86

Criterion validity

Criterion validity of MDAI-9 and MARS-M was established. The correlation between subscales of MDAI-9 and MARS-M is summarized in Table 4.

**Table 4:** Correlation between Subscale of MARS-M and MDAI-9 *Correlation is significant at the 0.05 level (1-tailed); **Correlation is significant at the 0.01 level (1-tailed) MDAI-9: Malay Translation of Drug Attitude Inventory 9, MARS-M: Medication Adherence Rating Scale-M

		MARS-M	
		Compliance Behaviour	Attitude towards medication
MDAI-9	Attitude towards medication	0.062 (p = 0.327)	.248^* ^(p = 0.035)
	Side effects	-.133 (p = 0.168)	-.330^**^ (0.007)

Mean score for MDAI-9

The sum of questions 1, 4, 6, 8 and 9 of the inventories (positive sum score) refers to symptom reduction. A sum score of < 13 and especially < 11 indicated that measures to improve attitude and compliance towards psychotropic medication are required. The mean positive sum score was 18.78 (SD 4.156). The sum of questions 2, 5 and 7 (negative sum score) refers to the presence of side effects of medication. The mean negative sum score was 5.78 (SD 2.353). A score of ≥ 7 suggests the presence of side effects to medication. The frequency of positive and negative sum scores is represented in Table 5.

**Table 5:** Frequency of positive and negative sum score

Items	N (%)
Positive Sum Score	
Desired attitude towards medication (score ≥ 14)	47 (87.2 %)
Undesired attitude toward medication (Score < 13)	7 (13.1%)
Negative Sum Score	
Presence of side effects (Score ≥ 8)	14 (25.9%)
No side effect (Score ≤ 7)	40 (74.1%)

## Discussion

The aim of this research was to create a Malay translation of the Drug Adherence Inventory 9 (MDAI-9) that is both valid and reliable. The MDAI-9 demonstrated strong internal consistency, with an overall Cronbach’s α for each factor ranging from 0.86 to 0.93. The content of MDAI-9 underwent a comprehensive review during the translation process, focusing on wording, sentence structure, and semantic accuracy. This review was conducted by experts in psychiatry proficient in both Malay and English languages. Face validity was established as all participants accurately understood the meaning of each item on the inventory.

The original DAI-9 has two factors [10]. Items 1, 4, 6, 8 and 9 formed the larger positive factor, which reflected the desired attitude to antipsychotic medications. Items 2, 5, and 7 constitute the negative factor that evaluates the presence of medication side effects. Item 3 was not found to load in any other factors in DAI-9. Exploratory factor analysis of MDAI-9 suggested a 2-factor solution. Item 3 showed high factor loading with other items in Factor 1, which consisted of items 1, 4, 6, 8 and 9. The items in the side effects factor (Items 2, 5, 7) showed high factor loadings, ranging from 0.776 to 0.86 indicating strong associations with the underlying construct.

Item 3, ‘I take medications of my own free choice’, was strongly associated with factor measuring the desired attitude to antipsychotic medications. Perceived autonomy in treatment played an important role in determining compliance with prescribed medications [15]. Studies have suggested that a sense of autonomy and involvement in treatment decisions were associated with an increased likelihood of adhering to medication regimens [16-17]. This sense of ownership can lead to a greater commitment to treatment goals and a better understanding of the benefits of medication. Studies have indicated that patients who perceive a high level of control over their health may exhibit psychological reactance when they perceive that their autonomy is being threatened by the treatment plan [17]. Collaborative decision-making between patients and healthcare providers could help patients feel empowered to participate in treatment decisions and therefore, were more likely to have the desired attitude towards antipsychotic medications [18-19]. This could explain why item 3 strongly represented that construct.

Analysis of MDAI-9 and MARS-M indicated a weak positive correlation between subscales measuring attitude to medications (0.248, p = 0.035). This suggested that the two-subscale measured similar construct and supports convergent validity. The subscale of MDAI-9 measuring side effects of medication showed a moderate negative correlation with the subscale of MARS-M measuring attitudes to medications. This correlation further confirmed valuable insights on the association between medication compliance behaviors and the underlying factors. This negative correlation suggested that individuals who experienced side effects of medication were less likely to have a desired attitude towards medication and, hence, a positive effect on adherence to the prescribed medication regimen. A study has reported early negative experiences with antipsychotic medication affected patients’ future decision-making around antipsychotic medications [20]. Understanding these correlations is important to help healthcare professionals identify beliefs and attitudes that influence medication adherence and steer targeted interventions to improve rates of medication compliance.

A large proportion of participants demonstrated a desired attitude towards medications. This was likely due to the local psychiatry operating policy in place that emphasized the importance of psychoeducation during consultation. This emphasis was also reflected in the annual audit of psychiatry done in all hospitals with psychiatry services where psychoeducation was included as mandatory during initial consultation and during hospitalization. Many studies have found that psychoeducational interventions had a beneficial impact on enhancing insight and promoting medication adherence in the majority of individuals with schizophrenia [21-22].

In many Asian cultures, family plays a central and crucial role in treatment decisions. Family’s perception and beliefs about psychiatry disorders and treatment influence patient’s medication compliance behaviors especially in chronic disorders such as schizophrenia [23-24]. Asians are close-knit communities that share strong bonds, common interests, and perceptions. Regrettably, this also influenced the dissemination of inaccurate information and misconceptions about psychiatric disorders. Therefore, family-linked and outreach programs focusing on educating families and communities about psychiatry disorders and treatment have been integrated into local psychiatry services development plans [25]. Furthermore, Talian Heal was introduced in 2022 as an initiative to provide the public with information on symptoms of major psychiatric disorders, lists of clinics and hospitals with psychiatry services in the country in addition to providing emotional and psychosocial support via tele-counseling [26]. 

The study was conducted in a multicultural setting. This allowed the effect of cultural diversity on the items in the questionnaire to be examined and therefore minimizing cultural bias. This helped the development of a culturally sensitive questionnaire that acknowledges the unique context of various cultures and is tailored to cultural differences. This ensures that the questionnaire is applicable in a local setting. 

The validation of MDAI-9 was conducted specifically in individuals with psychotic spectrum disorders, such as schizophrenia, schizophreniform disorder, brief psychotic disorder, and schizoaffective disorder. This narrow focus can limit the generalizability of the validation findings and restrict the questionnaire's applicability to disorders beyond psychotic conditions. Another important consideration is the limitation of a small sample size. While our study provides valuable insights, the generalizability of our findings may be limited. Future research with larger and more diverse samples is warranted. Additionally, participants in validation studies may have provided responses that they perceived as socially desirable, potentially skewing the accuracy of validity estimates away from reflecting their true experiences or behaviors.

## Conclusions

The Malay version of the Drug Adherence Inventory, known as MDAI-9, has shown good reliability and validity. It has been tested in a multicultural setting, thus making it generalizable across various cultures in a local setting. This makes it a valuable tool, particularly in Malaysia with its diverse cultural composition where there is a significant population who are fluent in Malay. The availability of MDAI-9 in Malay facilitates the assessment of medication adherence and attitude towards medication among Malay-speaking patients, enhancing its utility and relevance in clinical settings.
